# The Citation Wake of Publications Detects Nobel Laureates' Papers

**DOI:** 10.1371/journal.pone.0113184

**Published:** 2014-12-01

**Authors:** David F. Klosik, Stefan Bornholdt

**Affiliations:** Institute for Theoretical Physics, University of Bremen, Bremen, Germany; Technical University of Denmark, Denmark

## Abstract

For several decades, a leading paradigm of how to quantitatively assess scientific research has been the analysis of the aggregated citation information in a set of scientific publications. Although the representation of this information as a citation network has already been coined in the 1960s, it needed the systematic indexing of scientific literature to allow for impact metrics that actually made use of this network as a whole, improving on the then prevailing metrics that were almost exclusively based on the number of direct citations. However, besides focusing on the assignment of credit, the paper citation network can also be studied in terms of the proliferation of scientific ideas. Here we introduce a simple measure based on the shortest-paths in the paper's in-component or, simply speaking, on the shape and size of the *wake* of a paper within the citation network. Applied to a citation network containing *Physical Review* publications from more than a century, our approach is able to detect seminal articles which have introduced concepts of obvious importance to the further development of physics. We observe a large fraction of papers co-authored by Nobel Prize laureates in physics among the top-ranked publications.

## Introduction

To a certain degree science is a system of proliferation of ideas, since even the most innovative concepts emerge as answers to open problems and are based (at least to some extent) on previously established methods: They explain seemingly incompatible data within a new theoretic framework, or they make data accessible in the first place by rendering new measurements or calculations feasible. Fortunately, the associated book-keeping is done by scientists themselves due to the practice of citing the work they were influenced by in the reference list of their own publications. The aggregated citation information in a set of scientific publications therefore allows for deep insight in the structure and progress of science. As early as the 1960s it has first been depicted as a paper citation network, in which papers are represented by nodes and citations by directed links between the associated nodes of a citing paper and the corresponding cited paper [1], [2].

However, during the past decades, citation analysis has rather focused on the quantitative assessment of scientific research, interpreting citations as indicators of impact or assignment of credit [3], [4]. To this end, numerous quantitative measures have been developed [5], many of which are designed to rank scholars (e.g., the h-index [6]) or journals (e.g., the Thomson Scientific Journal Impact Factor). Until recently these measures were almost exclusively based on counting the number of a paper's direct citations (i.e., the respective node's in-degree), which is, indeed, known to have several shortcomings that have been discussed in the literature [7]–[10]. Motivated by the growing accessibility of citation databases, over the last decade some of these drawbacks have been addressed by a number of more elaborate impact measures that do not restrict themselves to mere direct citations (e.g., CiteRank [11], SARA [12], Eigenfactor [13]), but consider contributions to a paper's impact other than receiving the community's attention [14]. In particular, Chen *et al.* have shown that Google's PageRank algorithm [15], [16], that can be thought of as a diffusion process in which credit is equally distributed among the out-neighbours of a node, is able to find groundbreaking old papers (‘scientific gems’), that are rather moderately cited directly [17].

Focusing on the picture of idea propagation rather than of credit diffusion, we here introduce an approach to ranking scientific publications in a paper citation network that is based on an intuitive interpretation of the set of all publications that have cited a paper in question directly or indirectly (i.e., the paper's in-component).

## Results and Discussion

Let *G* be a paper citation network that is composed of the aggregated information about *m* citations between *n* papers, and let *i* be a paper that has been published at a point in time 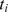. Clearly, paper *i* can only be cited by papers published at a later date 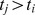, which again can receive citations only from papers published later than 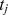 and so on, making *G* a directed acyclic graph (DAG). The wake-citation-score, 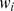, of a publication *i* is based on assigning all papers in *i*'s in-component, which we here call its *wake* 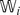, to *neighbourhood layers* 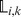 according to the length 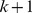 of the shortest-path(s) to *i* with 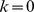 corresponding to the direct neighbours. Note that the resulting layers are disjoint and papers appear only in the closest one. The score 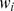 is then computed as a weighted sum over the cardinalities of the layers with the addends damped with a dilution factor depending on a parameter 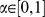, here chosen to be 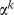. While for 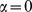 the computation is restricted to the zeroth layer, i.e., the direct citations, for 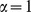 the whole wake is considered without any damping.

**Figure 1:**
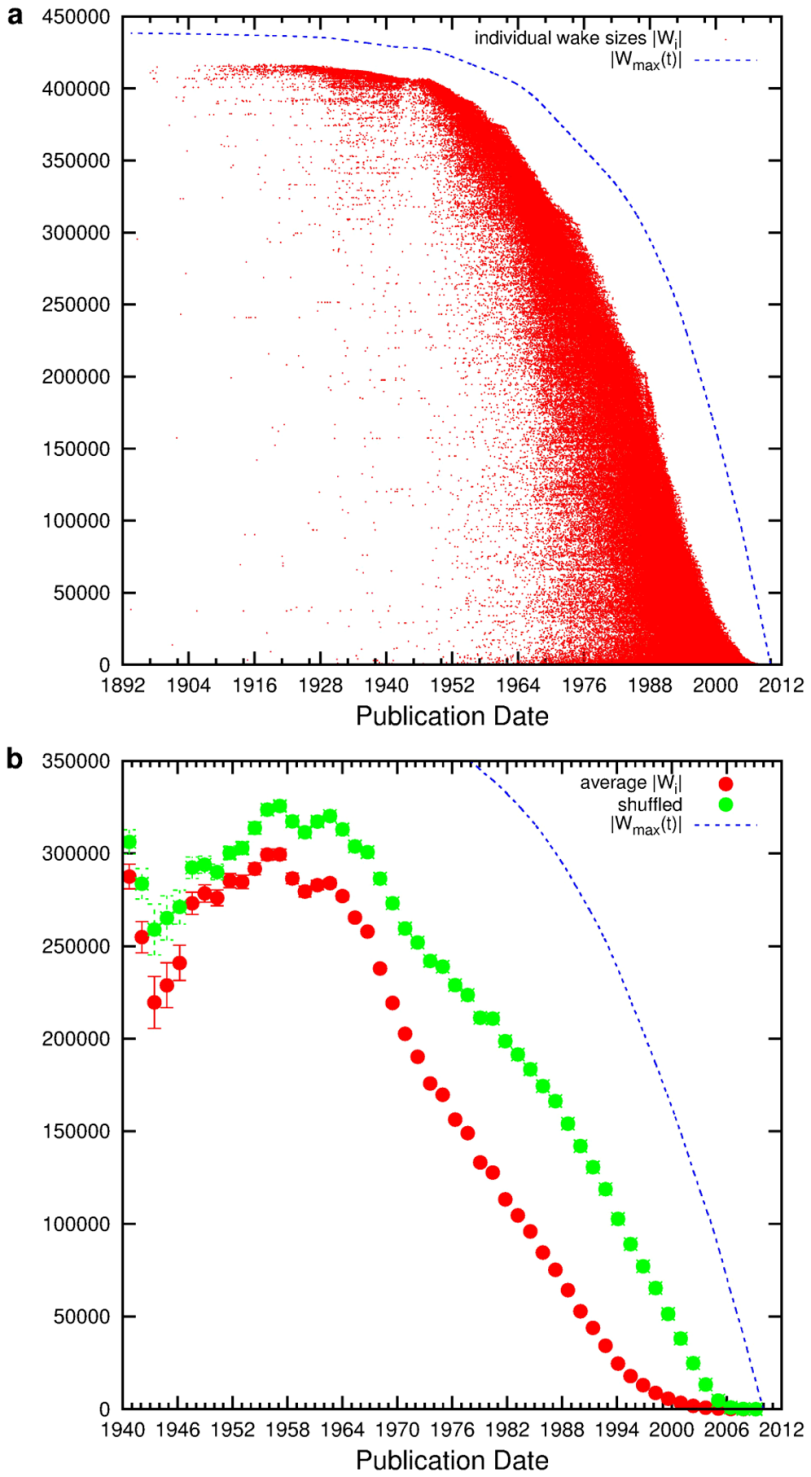
The papers' in-components. (**a**) The in-component sizes, 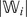, of all papers in the dataset are shown over the publication date. The dashed line depicts the size of the maximal in-component at a given publication date, 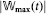. Early publications rarely show intermediate 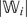 but are either rather poorly cited or have an in-component of about 90 percent of the maximum wake. The resulting ‘ridge’ formed by the data indicates cross-references between scientific subfields. For more recently published papers a subfield-structure is implied by the much smaller fraction of the maximum 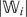 at a given time and the corresponding 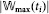. In (**b**) the data is averaged over time-windows of 500 days (red) and compared to wake-sizes obtained from a shuffled version (green) of the citation network, the shuffling being applied via a switching method that conserves the inherent time-arrow and the degree sequence of the network [27]. The link-shuffling leads to larger average in-component sizes for younger papers: the subfield-structure is not preserved in the shuffled network.

Although Fig. 1 shows that the total wake sizes yield information about the overall structure of the citation network (e.g., they indicate the presence of cross-references between different scientific subfields [18]), it is the case of 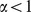 that reflects our notion of idea dilution in the network: The only explicit source of dilution in the wake-citation-score is given by the length of the shortest-path(s) between two papers, i.e., the minimal number of processing steps of an idea. In particular, the contribution to the score of a cited paper *i* is not normalized by the out-degree of the citing paper *j* (as it is the case in the PageRank algorithm), since this would correspond to reducing the influence of *i* on *j* due to the mere presence of other sources of ideas that *j* has used.

Eventually, a detrending is applied in order to address the fact that in a finite citation network there are the more possible citing papers the earlier a paper *i* has been published. We normalize the wake-citation-score by the size of the maximum possible in-component of a paper published at the time of *i*'s publication, 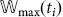, i.e., the number of all papers that are published after 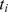 and arrive at the expression 
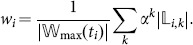
(1)


Note that this is a sum over disjoint layers of nodes emphasizing that the wake-citation-score counts weighted nodes and not weighted paths as centrality measures like PageRank do: Every paper 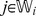 is evaluated exactly once in the computation of paper *i*'s score, while there is a contribution of *j* to the PageRank score of *i* for every directed path from *j* to *i* of which there can be many in the in-component of the paper *i*.

Since the assigning of disjoint neighbourhood layers corresponds to the construction of a shortest-paths-tree spanning the subgraph 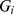 which contains all nodes in 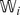 and the associated links, computing the wake-citation-scores for all nodes can be done by solving the all-pairs shortest-paths problem (APSP) and is thus of order 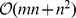 when a Breadth-First-Search is applied.

As mentioned above, the direct citation count is biased in several ways. In particular, it will strongly depend on the citation habits in the respective field and the size of the latter. Furthermore, within the period covered by the dataset used here (July 1893 - December 2009) there has been a remarkable growth of the scientific community as a whole. A paper published in the 1920s can potentially be cited by a much larger number of papers than a publication from the 1990s, but as the probability of being cited decays in time [19], one should also consider the number of papers published in the adjacent years when receiving citations is most likely: While there are not more than 1663 papers published in the whole 1920s in the dataset, from 1992 on in each single year more than 10000 papers are published. Also considering the fact that some seminal results have become textbook-knowledge, so that the original papers are no longer cited, the direct citation count might underestimate the impact of groundbreaking old publications. The wake-citation-score presented here is designed to address these issues.

**Figure 2:**
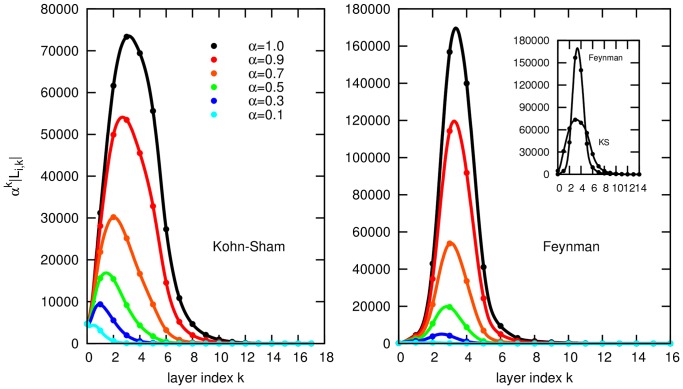
Neighbourhood layers of the citation wakes of two articles. The dilution concept in the computation of the wake-citation-score is illustrated by the cardinalities of the neighbourhood layers of two individual articles [20], [23]. In black the mere numbers of neighbours in the k-th layer are given (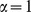), while other colours correspond to an actual dilution with 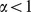. Note that the number of direct citations (at layer index 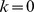) is large for the *Kohn-Sham*- but rather small for the *Feynman*-paper. The wake-citation-score for a specific *α* can be visually approximated by the area under the associated curve.

The three top-cited publications in the dataset are “*Self-Consistent Equations Including Exchange and Correlation Effects*” by W. Kohn and L. Sham [20], “*Inhomogeneous Electron Gas*” by P. Hohenberg and W. Kohn [21], and “*Self-interaction correction to density-functional approximations for many-electron systems*” by J. Perdew and A. Zunger [22] (cited 4728, 3682, 3169 times, respectively). Fig. 2 shows the individual wake of the Kohn-Sham paper. They all are seminal papers in the density-functional theory, the former two, indeed, prepared the ground for the method by introducing the Kohn-Sham equations and the Hohenberg-Kohn theorem. The number of direct citations seems to adequately represent their importance. But note the publication shown in the right frame of Fig. 2: In his 1949-paper “*Space-Time Approach to Quantum Electrodynamics*” R. Feynman introduced what became famous as the Feynman diagram [23]. Despite the undisputable fruitfulness of this concept the associated paper is rather moderately cited (206 times) and, considering direct citations only, is ranked #1031. While the citation count clearly underestimates its influence, applying the wake-citation-score yields one of the top-ranks (e.g., #6 for 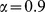, see below).

As a matter of course, there is no objective measure of importance or impact that the results of a bibliometric ranking scheme could be compared with. However, in order to argue that the wake-citation-score yields plausible results, in Table 1 we show the ten top-ranked papers according to their 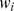-score with the parameter *α* chosen to be 0.9. Besides the aforementioned seminal publication by R. Feynman (ranked #6) the list is mainly composed of further groundbreaking papers whose high score is obviously plausible. Note for example the publication “*The Radiation Theories of Tomonaga, Schwinger, and Feynman*” in which F. Dyson shows the equivalence of Feynman's diagrammatic approach to quantum electrodynamics introduced in [23] with the formalism used by J. Schwinger and S.-I. Tomonaga. The concept of the Wigner-Seitz-cell, which has become common knowledge decades ago, was presented in the paper “*On the Constitution of Metallic Sodium*” by E. Wigner and S. Seitz in 1933, which is ranked #7 here. With the determinant representation of a basis of the anti-symmetrized fermionic many-body-wavefunction J. Slater had introduced an even more fundamental concept in 1929. His publication “*The Theory of Complex Spectra*” [24] is ranked #10 here. The remaining articles also seem properly placed within the top-ranked publications. However, except the highest ranked paper by Bardeen, Cooper and Schrieffer the ten top-ranked papers are far from being amongst the ten top-cited papers, in fact most of them are rather moderately cited and would not stand out remarkably in a direct citation-based ranking. The value given in the second column of Table 1 illustrates the wake-citation-score's intriguing feature of finding these articles: All shown publications show high ratios between the direct citation-rank and the rank assigned according to the wake-citation-score (Dyson's paper and Slater's “*The Theory of Complex Spectra*” are especially remarkable examples). Indeed, amongst the top-100 papers 86 show a ratio exceeding a value of 10.

**Table 1:** The ten top-ranked publications according to the wake-citation-score with dilution parameter *α* chosen to be 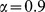.

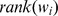	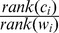	Publication	
1	10	Bardeen*, Cooper*, Schrieffer*	Theory of Superconductivity
2	707.5	F. Dyson	The Radiation Theories of Tomonaga, Schwinger, and Feynman
3	30.7	E. Wigner*	On the Interaction of Electrons in Metals
4	90.6	M. Gell-Mann*, K. Brueckner	Correlation Energy of an Electron Gas at High Density
5	18	S. Chandrasekhar*	Stochastic Problems in Physics and Astronomy
6	171.8	R. Feynman*	Space-Time Approach to Quantum Electrodynamics
7	166.9	E. Wigner*, F. Seitz	On the Constitution of Metallic Sodium
8	268.4	R. Feynman*	The Theory of Positrons
9	45.3	J. Slater	Atomic Shielding Constants
10	294.5	J. Slater	The Theory of Complex Spectra

Nobel Prize laureates are labelled with an asterisk (*), the *Feynman*-publication ranked #6 is also depicted in Fig. 2. The second column shows the fraction of the ranks assigned to the paper according to the number of direct citations and the wake-citation-score, respectively.

**Figure 3:**
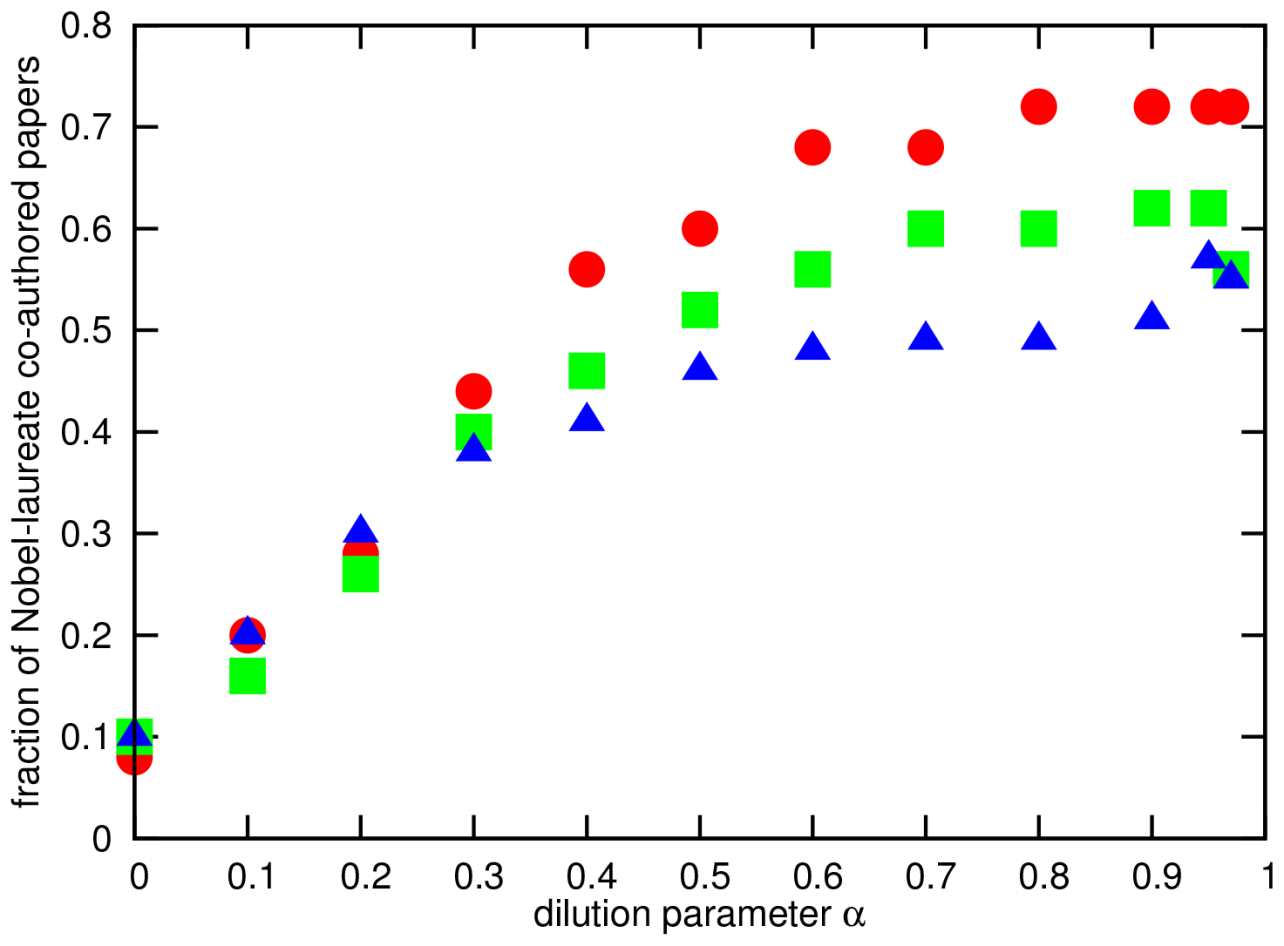
Fraction of Nobel-laureate co-authored papers. Fraction of papers with a co-author who has been awarded with a Nobel Prize in physics among the top-25 (red circles), top-50 (green squares) and top-100 (blue triangles) ranked papers according to the wake-citation-score for different dilution parameter values *α*.

As a second plausibility check of the outcome provided by the wake-citation-score we checked the top-ranked papers for the co-authorship of Nobel Prize laureates [25] in physics, which we consider to serve as a first-approximation proxy for both scientific quality and - although to a smaller extent, as there can be multiple papers co-authored by the same laureate in the list - diversity concerning physical subfields. In Table 1 the Nobel Prize laureates are labelled with a star. Fig. 3 illustrates the dependence on the dilution parameter *α*; since for a dilution parameter 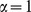 the wake-citation-score forfeits its ability to distinguish between different shortest-path lengths, i.e., different numbers of processing steps of an idea, we only show *α* values from the interval 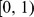. We found remarkable high fractions of papers co-authored by at least one physics Nobel Prize laureate among the 25, 50 and 100 top-ranked publications: e.g., for 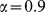 the list of the top-25 publications contains 18 of such papers (72%), 31 of them are among the top-raked 50, and still more than half of the top-100-ranked publications have been contributed to by a Nobel Prize laureate. This is in contrast to the ranking according to the direct citation count: not more than 4, 10 and 25 laureate-co-authored papers are among the top-25, top-50 and top-100 cited papers, respectively. We also compared our results to the outcome of a ranking obtained by the application of PageRank to the network as described in [17] and found that although PageRank also has more Nobel laureate co-authored papers among the top-ranked publications than the direct citation score, the wake-citation-score yields much higher values for a wide range of the dilution parameter *α* whose value is not critical and does not require tuning.

In summary, our approach of decomposing the papers' in-components into neighbourhood layers succeeds in finding groundbreaking publications that are rather moderately cited as well as in yielding a more diverse ranking than the direct citation count (which is dominated by the density-functional theory), without any explicit consideration of the lengths of the papers' reference lists and without an *a priori* assignment of the papers to specific subfields (as it is the case with relative indicators [26]).

## Materials and Methods

We apply the wake-citation-score to a paper citation network which is composed of papers published in the APS-journals between July 1893 and December 2009 and the associate citation information (which can be requested at http://journals.aps.org/datasets). Note that only internal citations (between APS-publications) are provided. In order to exclude publications that presumably show non-typical citation patterns, we restrict the citation network nodes to standard publications (which are tagged *article*, *brief*, *rapid* and *letter* in the APS-dataset) and reject material tagged *erratum*, *comment*, *reply*, *miscellaneous*, *editorial*, *announcement*, *essay*, *publisher-note*, *retraction*. The latter does not account for more than about 5 percent of all publications in the dataset. In addition, we discard self-links and citations that do not point backward in time, as well as parallel edges, and obtain an acyclic directed graph with 438296 nodes and 4560230 edges.
